# The genomic signature of resistance to platinum-containing neoadjuvant therapy based on single-cell data

**DOI:** 10.1186/s13578-023-01061-z

**Published:** 2023-06-08

**Authors:** Qihai Sui, Zhengyang Hu, Xing Jin, Yunyi Bian, Jiaqi Liang, Huan Zhang, Huiqiang Yang, Zongwu Lin, Qun Wang, Cheng Zhan, Zhencong Chen

**Affiliations:** Department of Thoracic Surgery, Zhongshan Hospital, Fudan University, 180 Fenglin Road, Xuhui District, Shanghai, 200032 China

**Keywords:** Neoadjuvant chemotherapy (NACT), Cisplatin (CDDP), Single-cell analysis, Gene expression, Drug resistance

## Abstract

**Background:**

Neoadjuvant chemotherapy (NACT) becomes the first-line option for advanced tumors, while patients who are not sensitive to it may not benefit. Therefore, it is important to screen patients suitable for NACT.

**Methods:**

Single-cell data of lung adenocarcinoma (LUAD) and esophageal squamous carcinoma (ESCC) before and after cisplatin-containing (CDDP) NACT and cisplatin IC50 data of tumor cell lines were analyzed to establish a CDDP neoadjuvant chemotherapy score (NCS). Differential analysis, GO, KEGG, GSVA and logistic regression models were performed by R. Survival analysis were applied to public databases. siRNA knockdown in A549, PC9, TE1 cell lines, qRT-PCR, western-blot, cck8 and EdU experiments were used for further verification in vitro.

**Results:**

485 genes were expressed differentially in tumor cells before and after neoadjuvant treatment for LUAD and ESCC. After combining the CDDP-associated genes, 12 genes, CAV2, PHLDA1, DUSP23, VDAC3, DSG2, SPINT2, SPATS2L, IGFBP3, CD9, ALCAM, PRSS23, PERP, were obtained and formed the NCS score. The higher the score, the more sensitive the patients were to CDDP-NACT. The NCS divided LUAD and ESCC into two groups. Based on differentially expressed genes, a model was constructed to predict the high and low NCS. CAV2, PHLDA1, ALCAM, CD9, IGBP3 and VDAC3 were significantly associated with prognosis. Finally, we demonstrated that the knockdown of CAV2, PHLDA1 and VDAC3 in A549, PC9 and TE1 significantly increased the sensitivity to cisplatin.

**Conclusions:**

NCS scores and related predictive models for CDDP-NACT were developed and validated to assist in selecting patients who might benefit from it.

**Supplementary Information:**

The online version contains supplementary material available at 10.1186/s13578-023-01061-z.

## Background

Although cancer incidence has declined at a steady pace since 2006–2007, malignant tumor, especially lung cancer, is the leading cause of cancer death in both men and women aged 50 years and older in the Global Cancer Statistics 2023 [1]. Similarly, according to the “Cancer Incidence and Mortality in China 2016” released by the National Cancer Center of China in 2022, about 4,064,000 new cancer cases and 2,413,500 new cancer deaths occurred in China in 2016 [2]. Cancer continues to be the leading cause of death and a significant barrier to increasing life expectancy in every country [3].

A new multimodal approach to cancer treatment began in the 1980s, starting with combination therapy in patients with locally advanced cancer. Rather than starting with the surgery, patients began with multiple courses of initial chemotherapy to determine tumor chemosensitivity and to reduce the burden of local and regional disease [4]. Neoadjuvant chemotherapy (NACT) has been proposed to be an essential treatment by some national and international guidelines of patients whose disease is at stages that warrant adjuvant chemotherapy, which aims to improve the efficacy of treatment in several advanced-solid-malignant-tumor patients [5, 6], because several randomized studies and meta-analysis showed that neoadjuvant chemotherapy combined with surgery significantly improved the long-term survival outcome of patients compared to surgery alone [7–11], while negative results occurred in unselected populations, limiting the widespread adoption of neoadjuvant therapy [9]. As a result, for those who are not sensitive to neoadjuvant therapy, neoadjuvant therapy delays surgery and may carry the risk of tumor progression.

Nowadays, neoadjuvant targeted therapy and immunotherapy have become new trends in preoperative adjuvant therapy for malignant tumors and have broad application prospects [6, 12–16]. However, owing to high price, uncertainty (still in clinical tails), and restriction (targeted and biologic therapies account for only 30% of all first-line treatments), platinum-based chemotherapy regimens remain a first-line treatment option or one of the components of neoadjuvant or adjuvant therapy for most of the cancers.

Therefore, further exploration of the molecular mechanisms of its progression is crucial for us to establish a particular neoadjuvant cisplatin evaluation, which enables the identification of treatment options associated with higher benefit or groups of patients with malignancies who specifically benefit from platinum-containing neoadjuvant chemotherapy.

## Methods

### Data processing and differential analysis

The single-cell analysis of LUAD and ESCC was conducted using the same methods described in our previous studies [17–19], which was approved by the Ethics Committee of Zhongshan Hospital, Fudan University (B2021–137R). To process the 10X genomics raw data, Cell Ranger software pipeline (version 3.0.0) was chosen. Alignment, filtering, barcode counting, and UMI counting were all carried out using the Cell Ranger in addition to demultiplexing raw base call files into FASTQ files. Patients had signed the informed consent at hospitalization. Differential genes were identified with P < 0.05 and false discovery rate (FDR) < 0.05 between the samples before and after neoadjuvant chemotherapy. They were also sorted according to logFoldChange values (|logFC|> 1) to identify significantly different expressions.

A previous study gained different genes associated with cisplatin sensitivity [20] based on details of cell lines information downloaded from Cancer Dependency Map (Depmap, depmap.org) and Cancer Cell Line Encyclopedia (CCLE, https://portals.broadinstitute.org/ccle/data), including IC50 value, cell line source, and mRNA expression. RNA-sequencing expression data and corresponding clinical information, especially the records of platinum-containing chemotherapy, were downloaded from the TCGA dataset (https://portal.gdc.com). The data processing process is similar to the previously published article. Survival differences were analyzed through the log-rank test. As the CCLE, GEO, and TCGA databases are open to the public under specific guidelines, it confirms that all written informed consents were obtained before data collection.

### GO, KEGG and GSVA analyses

GO analysis was performed to investigate the biological implications of proteins significantly associated with platinum response. R (version 3.6.1) was used for GSVA as well as GO and KEGG pathway enrichment analyses. The significance level was set to 0.05 for the corrected P-values. Bar maps and dot maps were used to visualize the consequences.

### Model contribution

The least absolute shrinkage and selection operator (LASSO) regression algorithm, which obtains a more refined model by constructing a penalty function that makes it compress some coefficients while setting some coefficients to zero, was used for feature selection, tenfold cross-validation was used, and the R package glmnet was used for the analysis.

All the analysis methods and R packages regarding single cell were implemented by R (foundation for statistical computing 2020) version 4.0.3 and the others were implemented by R version 3.6.1. P-value < 0.05 was considered statistically significant.

### Survival analysis

K-M plot was conducted through (https://kmplot.com/analysis/).

Univariate and multivariate cox regression analyses were performed to identify the proper terms to build the nomogram. The forest was used to show each variable’s P value, HR and 95% CI through ‘forestplot’ R package. A nomogram was developed based on the results of multivariate cox proportional hazards analysis to predict the X-year overall recurrence. The nomogram provided a graphical representation of the factors which can be used to calculate the risk of recurrence for an individual patient by the points associated with each risk factor through ‘rms’ R package.

### Cell culture and cytotoxic assay

LUAD A549 and PC9 cells, ESCC TE1 cells were obtained from the American Type Culture Collection (Manassas, VA, USA). Cells were fostered in DMEM containing 10% fetal bovine serum (FBS) and 100 μg/mL of penicillin–streptomycin with or without CDDP (Sigma-Aldrich, Merck KGaA, Darmstadt, Germany) added into the culture medium for incubation in a humid atmosphere containing 5% CO2 at 37 °C.

Cell proliferation was evaluated by Cell Counting Kit-8(CCK-8; Dojindo, Kumamoto, Japan) and EdU Cell Proliferation Kit (E607204, Sangon Biotech, Shanghai, China). Briefly, for CCK-8, 2 × 10^3^ of A549, PC9 and TE1 cells were plated in 96 well plates and were incubated with 100 μL DMEM containing 10% fetal bovine serum (FBS) and 100 μg/mL of penicillin–streptomycin for 24 h, then with or without CDDP (Sigma-Aldrich, Merck KGaA, Darmstadt, Germany) for another 24 h at 37 °C. After treatment, cells were incubated in 10% CCK-8 reagent. The OD value was measured after 2 h at 450 nm with a microplate reader from Bio-Rad (Microplate reader 3550-UV).

For EdU, 2.5 × 10^5^ of A549, PC9 and TE1 cells were plated in 24 well plates and were incubated with 200 μL DMEM containing 10% fetal bovine serum (FBS) and 100 μg/mL of penicillin–streptomycin for 24 h, then with or without CDDP (Sigma-Aldrich, Merck KGaA, Darmstadt, Germany) for another 24 h at 37 °C. After treatment, cells were cultured in 10 uM EdU for 2 h. Then incubation for 30 min at room temperature with 4% paraformaldehyde cell fixative, followed by 0.5% Triton X-100 cell permeabilizing solution for 10 min at room temperature. After that, Using TAMRA red fluorescent solution incubate for 30 min at room temperature, then add Hoechst staining solution and incubate for 20–30 min at room temperature and avoid light. Immediately after staining, observation through fluorescence microscopy was performed.

### RNA interference

siRNAs targeting PHLDA1, CAV2, VDAC3, and Silencer Negative Control siRNAs were purchased from Ribobio (sequences provided in Additional file 1: Table S1). We purchased 2 different siRNAs for each gene to avoid the off-target effects. A549, PC9 and TE1 cells were seeded in 6-well plates for 24 h prior to transfection with siRNA targeting PHLDA1, CAV2, VDAC3 and corresponding non-targeting controls. A total of 180 nM of siRNA was added to each experiment made up of the target siRNA and topped up with the appropriate concentration of non-targeted controls where appropriate. Transfections were carried out in OptiMem medium (Gibco) using Lipofectamine 8000 transfection reagent (Beyotime). 48 h post-transfection cells were harvested and assayed for RNA and protein expression levels of the target of interest. At the same time, corresponding samples were treated as described in the text.

The sequences of siRNAs were listed in Additional file 1: Table S3.

### RNA preparation and qRT-PCR analysis

To detect the expression of PHLDA1, CAV2, VDAC3 in A549, PC9 and TE1 cell lines, RT-qPCR was carried out on an ABI Prism 7500 real-time PCR system (Applied Biosystems) with proper PCR parameters.

The steps and the reagents used for qRT-PCR were the same as the previous study. β-actin was used as the reference [20]. Primers used in this study are listed in Additional file 1: Table S2.

All the samples were repeated 3 times.

### Western blot analysis

Proteins of A549, PC9 and TE1 cells after RNA interference were extracted using RIPA (Beyotime, Shanghai, China) with protease and phosphatase inhibitor cocktail (Beyotime). The steps and the reagents used for measuring protein concentration and western blot are as described in previous studies [21].

Finally, we observed the protein bands with the Moon chemiluminescence kit (Beyotime). The following antibodies were used: Rabbit anti-CAV2 (CY5010, dilution 1:1500, Abways); Rabbit anti-PHLDA1 (AY3597, dilution 1:1500, Abways); rabbit anti-VDAC3 (55260-1-AP, dilution 1: 1500, Proteintech), mouse β-ACTIN (1:3000, AA128, Beyotime), horseradish peroxidase (HRP)-labeled goat anti-rabbit IgG (H + L) (1:3000, A0208, Beyotime), and HRP-labeled goat anti-mouse IgG (H + L) (1:3000, A0208, Beyotime).

All the samples were repeated 3 times.

## Results

### Single-cell analysis of differentially expressed genes in tumor cells before and after neoadjuvant therapy

**Fig. 1:**
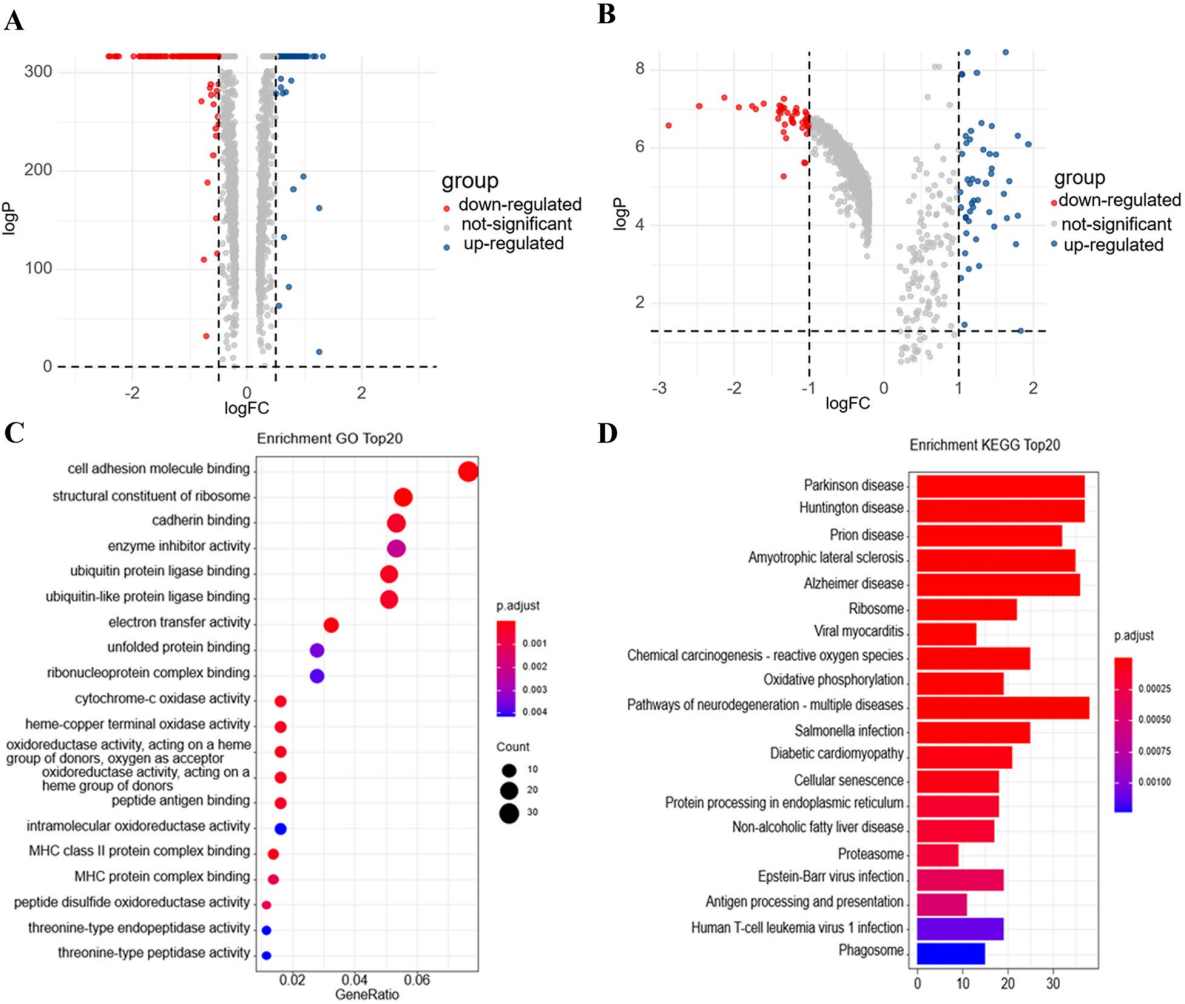
differential analysis for tumor cells before and after neoadjuvant therapy treated with cisplatin-containing neoadjuvant chemotherapy. **A**: Differentially expressed genes before and after neoadjuvant chemotherapy in patients with LUAD. **B**: Differentially expressed genes before and after neoadjuvant chemotherapy in patients with ESCC. **C**: Dot plot showing results of GO analysis applied to combined differentially expressed genes of LUAD and ESCC; **D**: Bar plot showing results of KEGG analysis applied to combined differentially expressed genes of LUAD and ESCC

Firstly, we selected tumor cells before and after neoadjuvant chemotherapy in patients with lung adenocarcinoma (LUAD) and esophageal squamous carcinoma (ESCC) treated with cisplatin-containing neoadjuvant chemotherapy, respectively, and performed differential analysis. These differential genes were intersected to obtain 485 genes expressed differentially in tumor cells before and after neoadjuvant treatment for LUAD and ESCC. The differential analysis of GO functional enrichment of these 485 genes revealed that they mainly focused on cell adhesion molecule binding, enzyme inhibitor activity, cadherin binding. KEGG signaling pathway enrichment revealed that they mainly focus on metabolites, degenerative changes, carcinogenesis, etc. Fig. 1.

### Combined analysis of neoadjuvant chemotherapy differential genes and cisplatin resistance-associated genes

**Fig. 2:**
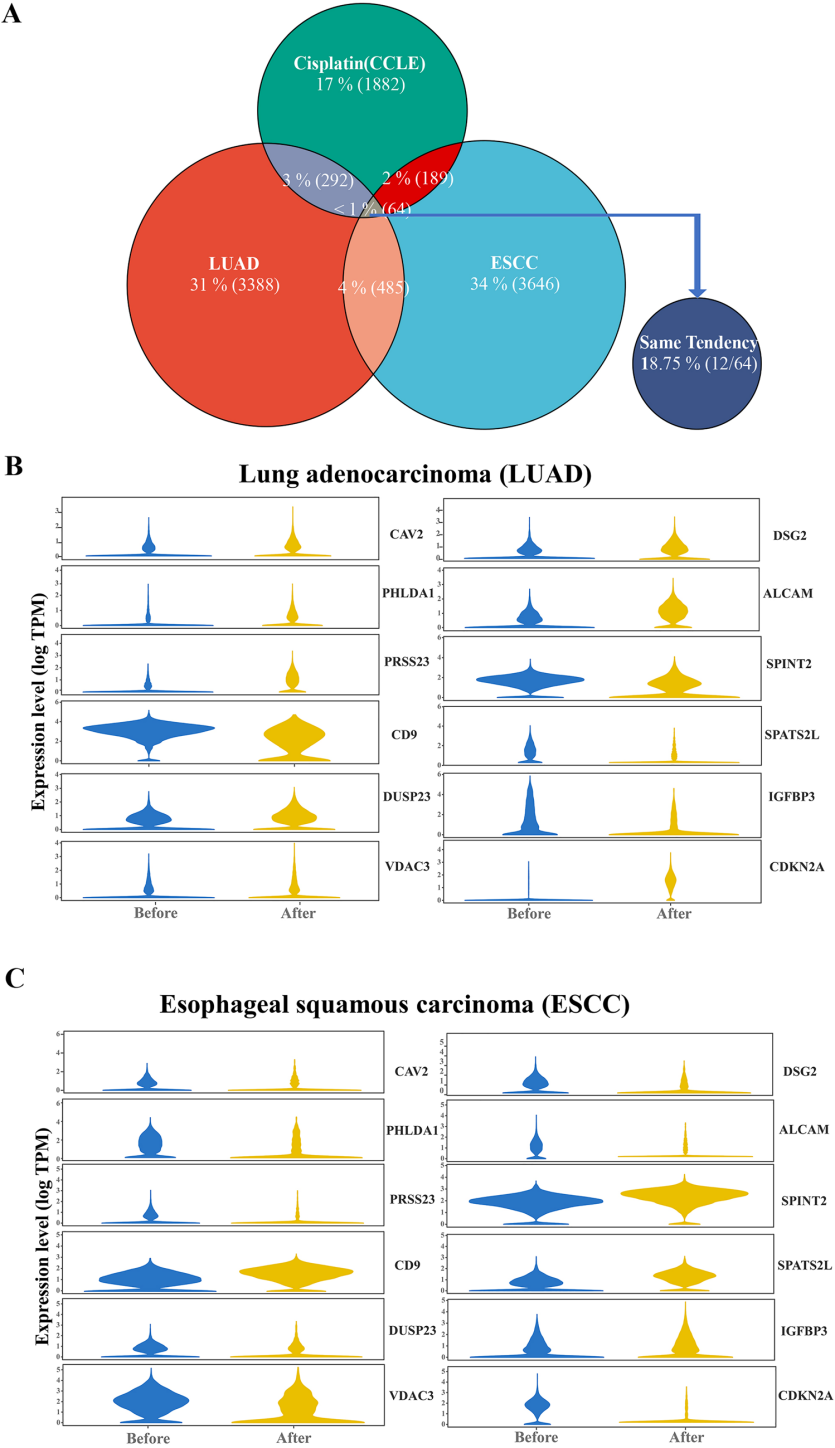
**A**: Venn diagram showing the intersection of differential genes with consistent expression trends in residual tumor cells after neoadjuvant chemotherapy for LUAD, ESCC, and cisplatin-resistant cell lines; **B**: Expression of 12 genes in tumor cells before and after neoadjuvant therapy for lung adenocarcinoma; **C**: Expression of 12 genes in tumor cells before and after neoadjuvant therapy for ECC

In our previous study, we performed a multi-omics analysis of cisplatin-resistant and sensitive cell lines, in which we obtained a relevant differential mRNA set. We intersected this mRNA set and the 485 differential genes obtained previously and reached the result of 64 critical genes associated with neoadjuvant chemotherapy efficacy. These 64 genes were eliminated one by one. Finally, 12 genes with consistent expression trends in both LUAD, ESCC residual tumor cells after neoadjuvant chemotherapy and cisplatin-resistant cell lines were found (Fig. 2) (Additional file 1: Table S1).

### Validation of neoadjuvant chemotherapy score (NCS) in LUAD, ESCC single-cell sequencing samples before and after NACT as well as CCLE database

**Fig. 3:**
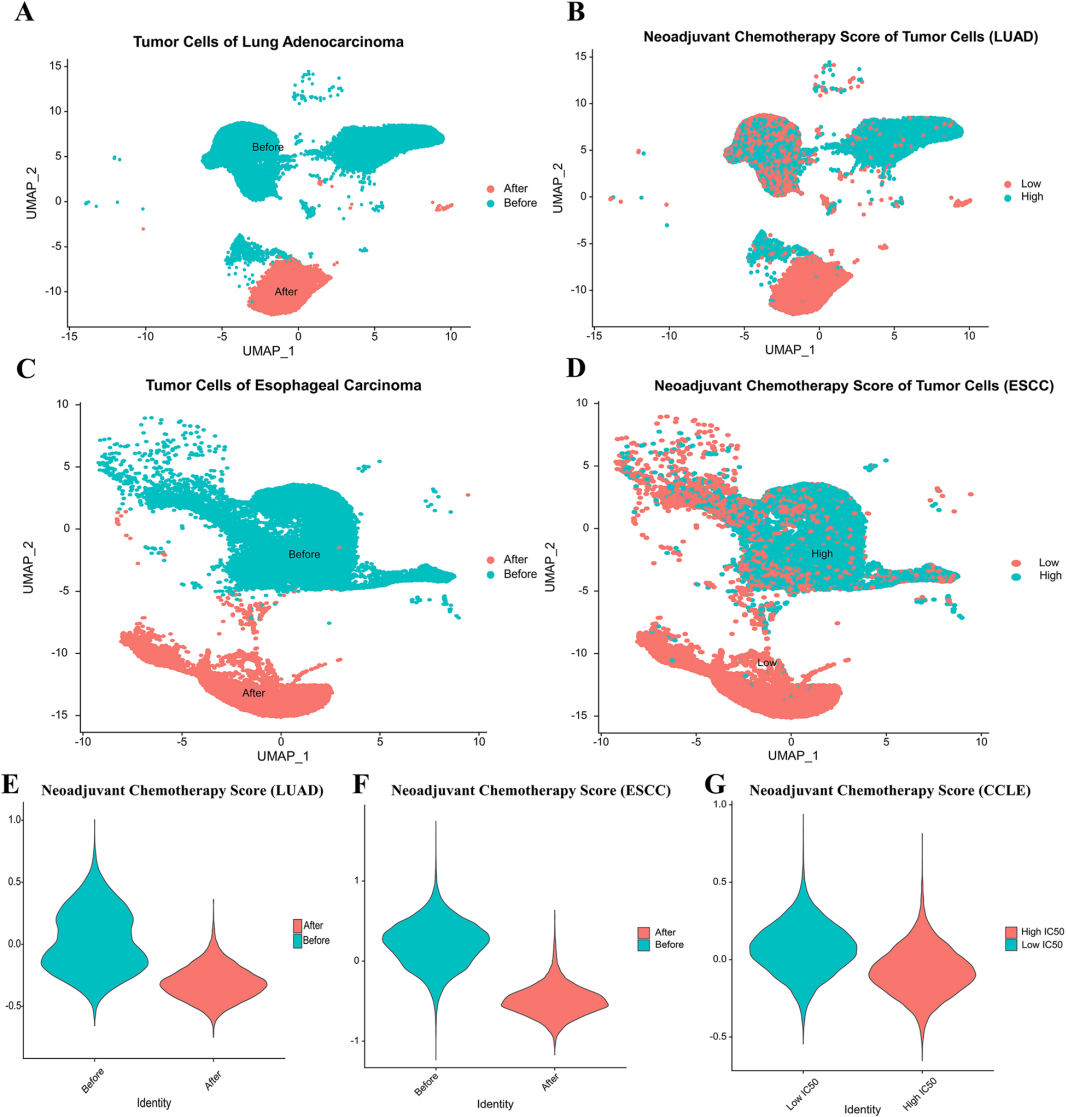
**A**, **C**: Single cell data of LUAD (A) and ESCC (C) tumor cells before and after NACT were separated according to UMAP (Uniform Manifold Approximation and Projection); **B**, **D**: Tumor cells of LUAD (B) and ESCC (D) were divided into two groups of high and low NCS by the mean value of NCS; **E**, **F**: Violin plot showing NCS values of LUAD (E) and ESCC (F) tumor cells before and after NACT; **G**: Violin plot showing NCS values in high and low cisplatin IC50 groups

We reintroduced the 12 genes, CAV2, PHLDA1, DUSP23, VDAC3, DSG2, SPINT2, SPATS2L, IGFBP3, CD9, ALCAM, PRSS23, CDKN2A, constructed as Neoadjuvant Chemotherapy Score into the single cell sequencing data of LUAD and ESCC patients before and after neoadjuvant therapy as well as the CCLE database. It was not difficult to find that the NCS of tumor cells before neoadjuvant therapy was significantly higher than that of tumor cells after neoadjuvant therapy in LUAD and ESCC (Fig. 3A–F). The NCS in the low IC50 group, which is the cisplatin-sensitive group, was higher than that in the high IC50 group in the CCLE database (Fig. 3G).

### Application of 12 genes and NCS in LUAD and ESCC single-cell sequencing samples

**Fig. 4:**
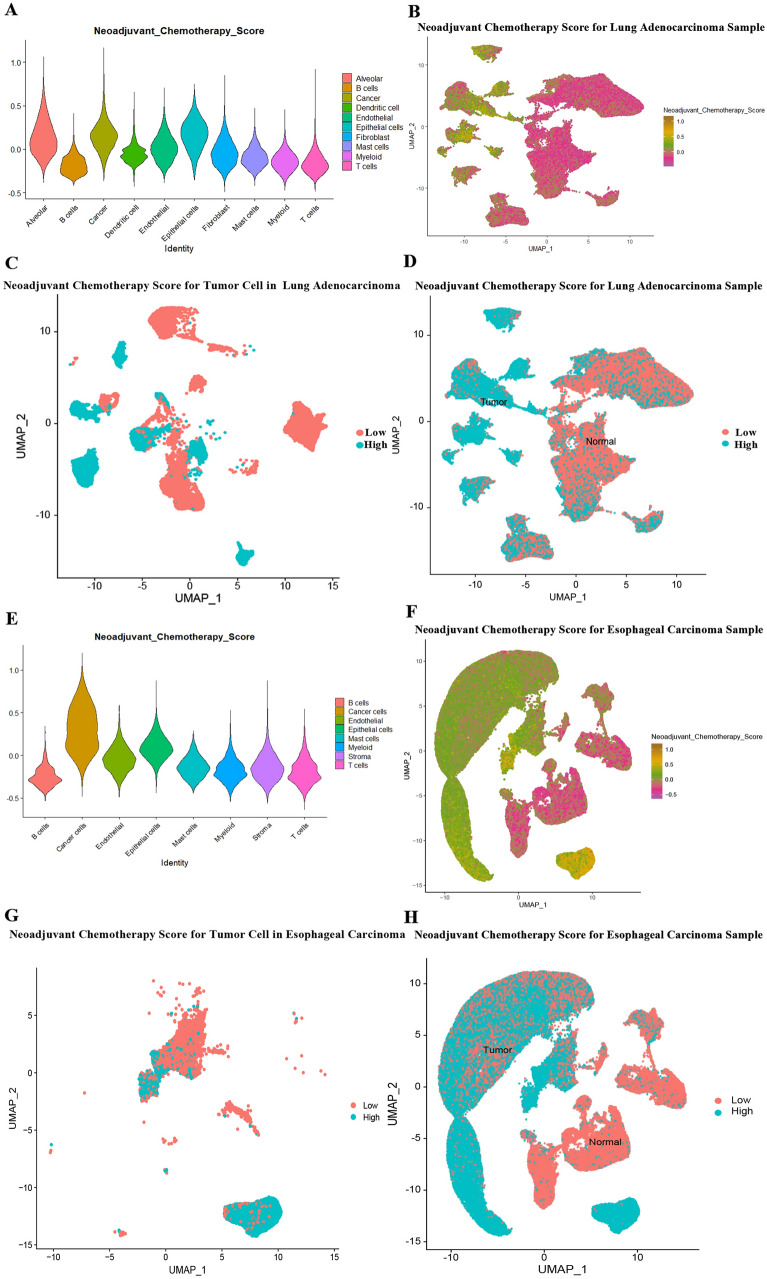
**A**, **E**: Violin plot showing the NCS values of each cell subpopulation in LUAD (A) and ESCC (E) tumors and normal tissues beside the tumors; **B**, **F**: UMAP plot showing the high and low NCS values of each cell in LUAD (B) and ESCC (F) tumors and normal tissues beside the tumors; **C**, **G**: Separating LUAD (C) and ESCC (G) tumor cells into two groups of high and low NCS; **D**, **H**: Tumor and peri-tumor normal tissues were separated according to UMAP, and all cells of LUAD (C) and ESCC (G) were divided into two groups according to the average value of NCS

Analyses were then performed on single-cell sequencing samples obtained from LUAD patients. Tumor cells were selected to detect the expression of the above 12 genes, and it was not difficult to find that CAV2, PHLDA1, and VDAC3 were generally highly expressed in tumor cells. (Additional file 2: Fig S1). NCS was then constructed using the AddModuleScore function in R to show the overall expression of the 12 genes in different cell subgroups in the overall LUAD, ESCC and the corresponding paraneoplastic tissue samples (Fig. 4A, E). The samples were also classified by tumor and normal tissue. It was not difficult to find that the NCS, compared to the expression of the 12 genes, was significantly higher in tumor tissue than in normal tissue (Fig. 4B, D, F, H). Further, in tumor cells, we applied the NCS. The tumor cells were divided into 2 groups according to the mean values to further investigate their differences (Fig. 4C, G).

### Analysis of differentially expressed genes in tumor cells grouped by NCS

**Fig. 5:**
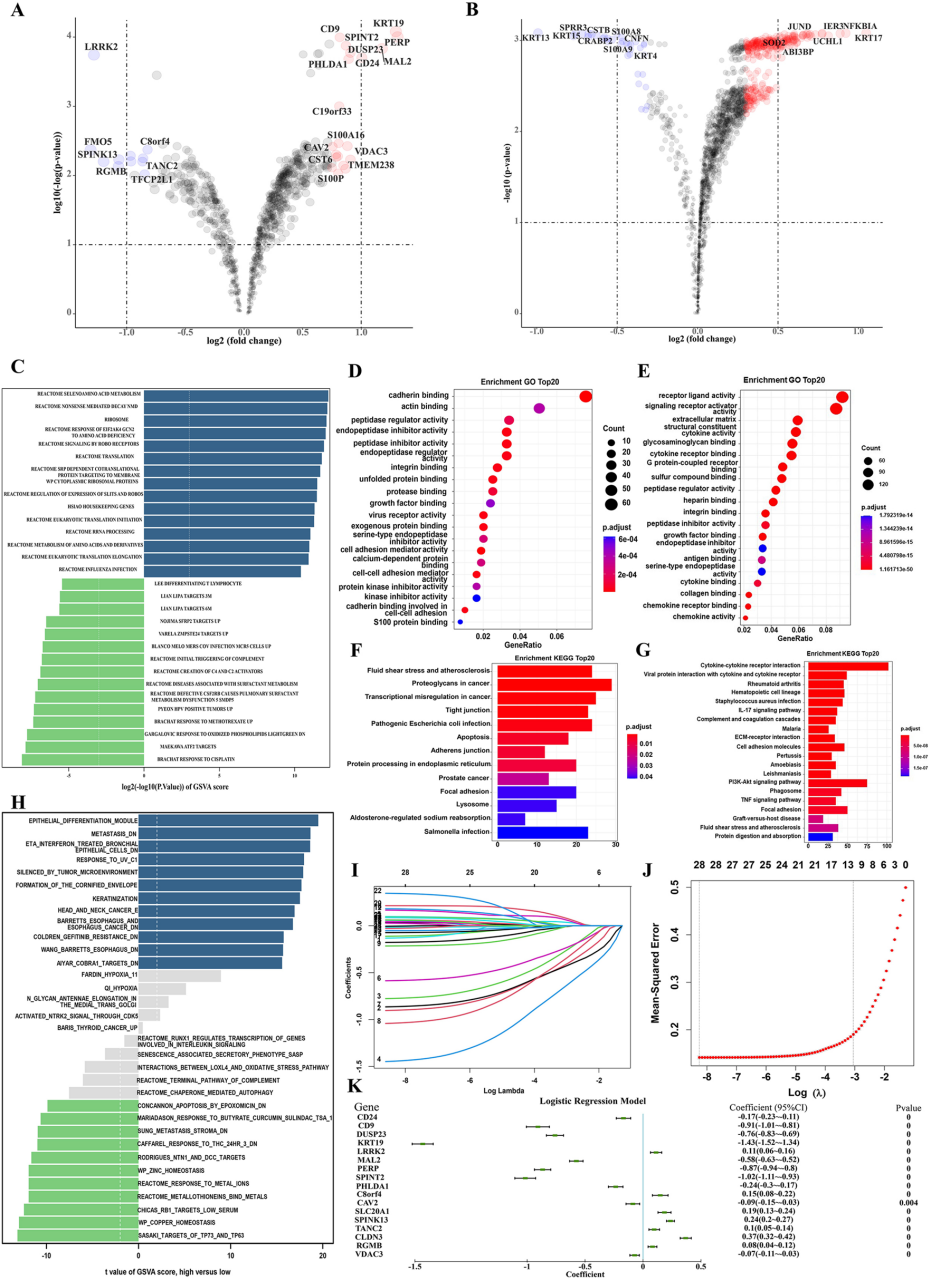
**A**, **B**: Volcano maps for differentially expressed genes in LUAD (A) and ESCC(B); **D**, **E**: Dot plot for GO analysis in LUAD (D) and ESCC (E); **F**, **G**: Bar plot for KEGG analysis in LUAD (F) and ESCC (G); **C**, **H**: Bar plot for GSVA analysis in LUAD (C) and ESCC (H); **I**, **J**: Establishment of the LASSO model relating to cisplatin sensitivity in cancer cells; **K**: Coefficient display of logistics regression equation model of cisplatin-sensitivity related genes

Then we applied the NCS on single-cell sequencing samples obtained from LUAD and ESCC patients and divided tumor cells into two groups. In LUAD, we then performed differential analysis between these two groups and obtained a total of 796 differentially expressed genes (p < 0.001), of which 119 had significant fold change (|logFC|> 0.5). 77 of these 119 genes were significantly up-regulated in the low NCS group, including VDAC3, CAV3, PHLDA1, CD9, etc., and 42 were significantly downregulated, including LRRK2, SPINK13, TANC2, etc. (Fig. 5A). The GO functional enrichment analysis of these 119 significantly differentially expressed genes revealed that they were mainly concentrated in cadherin binding, actin binding, peptidase regulator activity, endopeptidase inhibitor activity, etc. (Fig. 5D), and the KEGG signaling pathway enrichment revealed that they were mainly concentrated in fluid shear stress and atherosclerosis, proteoglycans in cancer, transcriptional misregulation in cancer, etc. (Fig. 5F). We then performed further differential analysis by GSVA at the overall level of gene function and signaling pathways. We found that seleno amino acid metabolism, nonsense mediated decay nmd, ribosome, response of EIF2AK4 GCN2 to amino acid deficiency were significantly up-regulated in the high NCS group. In contrast, ATF2 targets, response to oxidized phospholipids, and response to methotrexate were significantly downregulated (Fig. 5C).

Similarly, in ESCC, we obtained 1676 differential genes, 65 significantly up-regulated by KRT17, NFKBIA, IER3 and 14 significantly down-regulated by KRT13, KRT15, SPRR3, etc. (Fig. 5B). Different GO relied on receptor-ligand activity, extracellular matrix, etc. (Fig. 5E). The KEGG signaling pathways were enriched in Cytokine-cytokine receptor interaction, Viral protein interaction with cytokine and cytokine receptor, IL-17 signaling pathway, etc. (Fig. 5G). GSVA found that Tumor microenvironment, Epithelial cells, as well as Barrett's esophagus and esophagus cancer were significantly up-regulated in the high NCS group, while TP73 and TP63 targets, Response to metal ions, Response to THC were significantly downregulated (Fig. 5H).

And then, we performed LASSO regression analysis on the expression data of the intersection of two sets of differential genes and obtained the coefficients and binomial deviation curves in LASSO regression with log(λ) (Fig. 5I, J). The predictive effects of the models of the obtained characteristic variables were different for different λ taking values. The 22 characteristic variables, CD24, D9, DUSP23, KRT19, LRRK2, MAL2, PERP, SPINT2, TACSTD2, PHLDA1, C8orf4, CAV2, CAPN8, MDK, SLC20A1, SPINK13, TANC2, CLDN3, RGMB, S100P, VDAC3, and C19orf33 that minimized the mean squared deviation achieved by the whole model were selected to construct the prediction model. We then formed a generalized linear equation by glm function for these 22 genes. 17 indicators were obtained to be enrolled and a model: score = (− 0.17) *CD24 + (− 0.91) *CD9 + (− 0.76) * DUSP23 + (− 1.43) *KRT19 + 0.11*LRRK2 + (− 0.58) *MAL2 + (− 0.87) *PERP + (− 1.02) *SPINT2 + (− 0.24) *PHLDA1 + 0.15 *C8orf4 + (− 0.09) *CAV2 + 0.19 *SLC20A1 + 0.24 *SPINK13 + 0.1*TANC2 + 0.37*CLDN3 + 0.08 *RGMB + (− 0.07) *VDAC3, was constructed to predict the high and low NCS (Fig. 5K).

### Survival analysis of 12 genes among LUAD patients with chemotherapy records

Based on the TCGA and GEO datasets, we first performed a K-M survival analysis for each gene in LUAD patients with chemotherapy records. As described in Additional file 2: Fig S1, these genes, CAV2, PHLDA1, ALCAM, CD9, IGBP3, and VDAC3, were significantly associated with prognosis (p < 0.05). Except for ALCAM, the prognosis of patients with high expression of the other five genes was worse. The most significant association with prognosis was observed for CAV2, PHLDA1 and VDAC3.

**Fig. 6:**
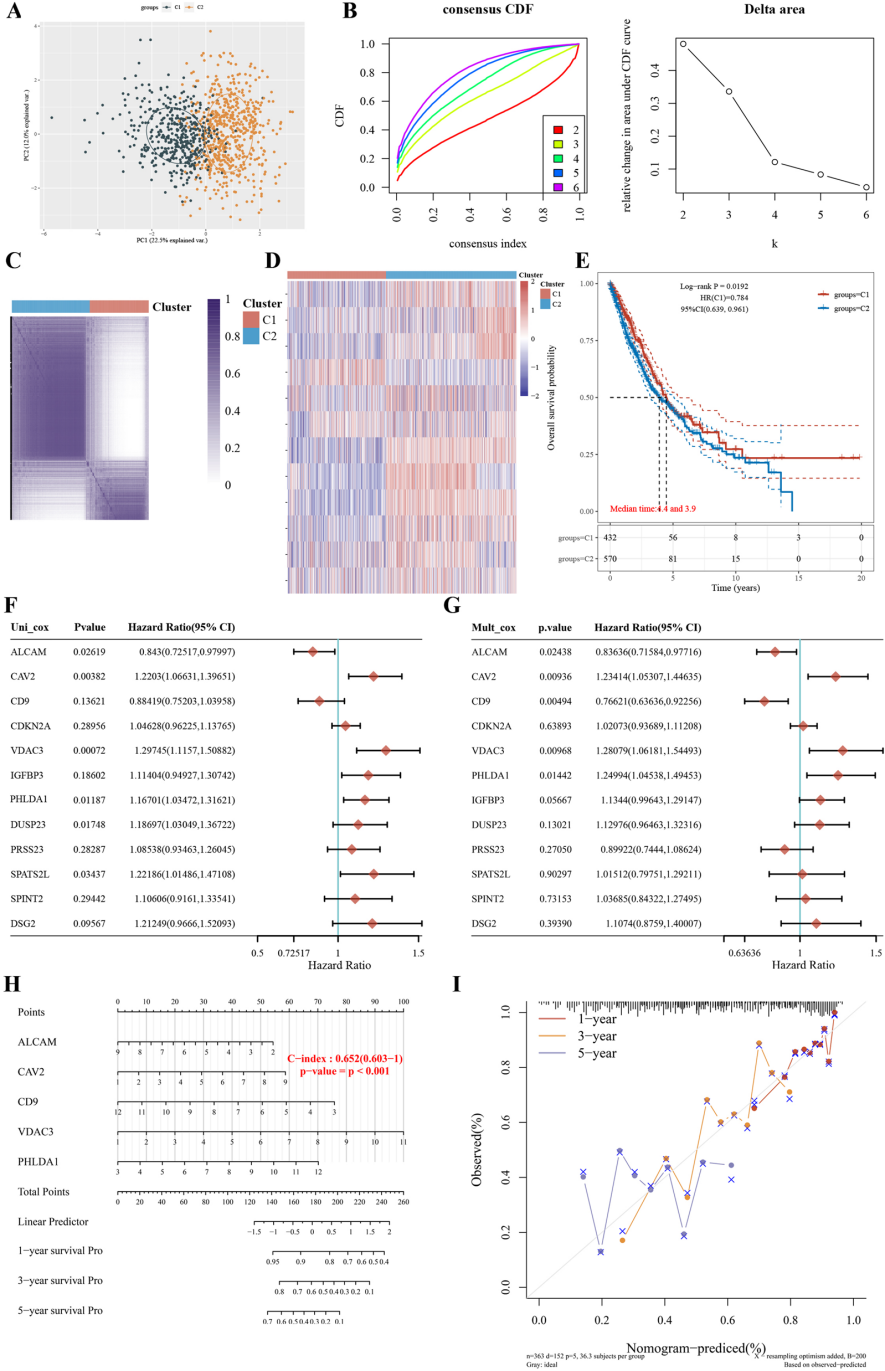
**A**: The ConsensusClusterPlus consistency clustering scatter plot at k = 2; **B**: The consistency clustering curve (CDF) and the CDF Delta area curve; **C**: The ConsensusClusterPlus consistency clustering heat map at k = 2, which can show the two categories with obvious differences; **D**: Rows and columns represent samples, different colors represent different categories, and the heat map of the expression of 12 related genes in 2 subgroups, red represents high expression and blue represents low expression; **E**: Kaplan–Meier survival analysis of the 2 groups of samples from TCGA dataset, comparison among different groups was made by log-rank test. HR (95%Cl), the median survival time (LT50) for different groups. **F**–**I**: Contribution and validation of nomogram model. The p-value, risk coefficient (HR) and confidence interval are analyzed by univariate (F) and multivariate (G) Cox regression. Nomogram can predict the 1 year, 2 year and 3 year overall survival of LUAD patients with chemotherapy (H). Calibration curve for the overall survival nomogram model in the discovery group. The dashed diagonal line represents the ideal nomogram, and the blue line, red line and orange line represent the 1 year, 2 year and 3 year of the observed nomogram (I)

We further wanted to validate and explore the probable mechanism in all LUAD patients. We then used these 12 genes for clustering analysis in LUAD patients in the TCGA database. We found that at k = 2 these genes could clearly classify patients into two subtypes (Fig. 6A–D). There was a significant survival difference between these two subtypes (Fig. 6E), so we could tentatively conclude that in LUAD, by the expression of these 12 genes, we could get a platinum-containing adjuvant chemotherapy insensitive subtype, suggesting that patients in this subtype may not benefit from neoadjuvant or adjuvant chemotherapy.

Furthermore, cox and nomogram analyses were performed based on these 12 genes. Univariate analysis (Fig. 6F) revealed that ALCAM, CAV2, VDAC3, PHLDA1, DUSP23, and SPATS2L were significant predictors of the prognosis of LUAD patients, Multivariate Cox proportional hazard analysis (Fig. 6G) demonstrated ALCAM, CAV2, CD9, VDAC3, PHLDA1 were independent prognostic factors for survival in LUAD patients. A nomogram relating to 5 independent risk factors (ALCAM, CAV2, CD9, VDAC3, PHLDA1), which were concluded from MVA. The Points could calculate 1 year, 3 year and 5 year overall survival (OS) at the model’s top (Fig. 6H). The internal evaluation was performed (Fig. 6I) with the same TCGA database, the C-index of which was 0.652 (0.603–1). In general, LUAD patients with a lower expression of CAV2, VDAC3, and PHLDA1 had longer predicted survival time.

### Further validation in TCGA database

Besides lung adenocarcinoma, we selected 2 cancer types in the TCGA database that contain cisplatin in the standard neoadjuvant therapy and have chemotherapy records: gastric adenocarcinoma and head and neck squamous carcinoma. The genes previously obtained were subjected to LASSO regression analysis to initially screen for genes that might be associated with survival in neoadjuvant patients and then build the corresponding logistic regression models (Additional file 4: Fig S3A: gastric adenocarcinoma; 3B head and neck squamous carcinoma), with the model obtained for gastric adenocarcinoma the model was obtained as Risk score = (0.0339) * CAV2 + (0.0237) * DUSP23 + (− 0.0128) * VDAC3 + (0.0281) * SPATS2L + (0.0779) * PRSS23; the model obtained for squamous carcinoma of the head and neck was: Risk score = (0.041) * CAV2 + (0.0359) * PHLDA1 + (0.05) * DSG2 + (0.0298) * ALCAM + (0.0221) * PRSS23 + (− 0.0394)* CDKN2A. The models were then validated separately, firstly, the distribution of KM survival curves, and it was found that there were significant survival differences between the two groups into which the models divided the dataset, secondly, the ROC curves and AUCs of the risk models at different times were depicted, and the AUCs of the three models at 1, 3 and 5 years were all greater than 0.6, obviously, higher AUC values indicates the stronger predictive ability of the model (Additional file 4: Fig S3C, D).

### Expression of PHLDA1, CAV2, VDAC3 is associated with the sensitivity of cisplatin in A549, PC9 and TE1 cells

**Fig. 7:**
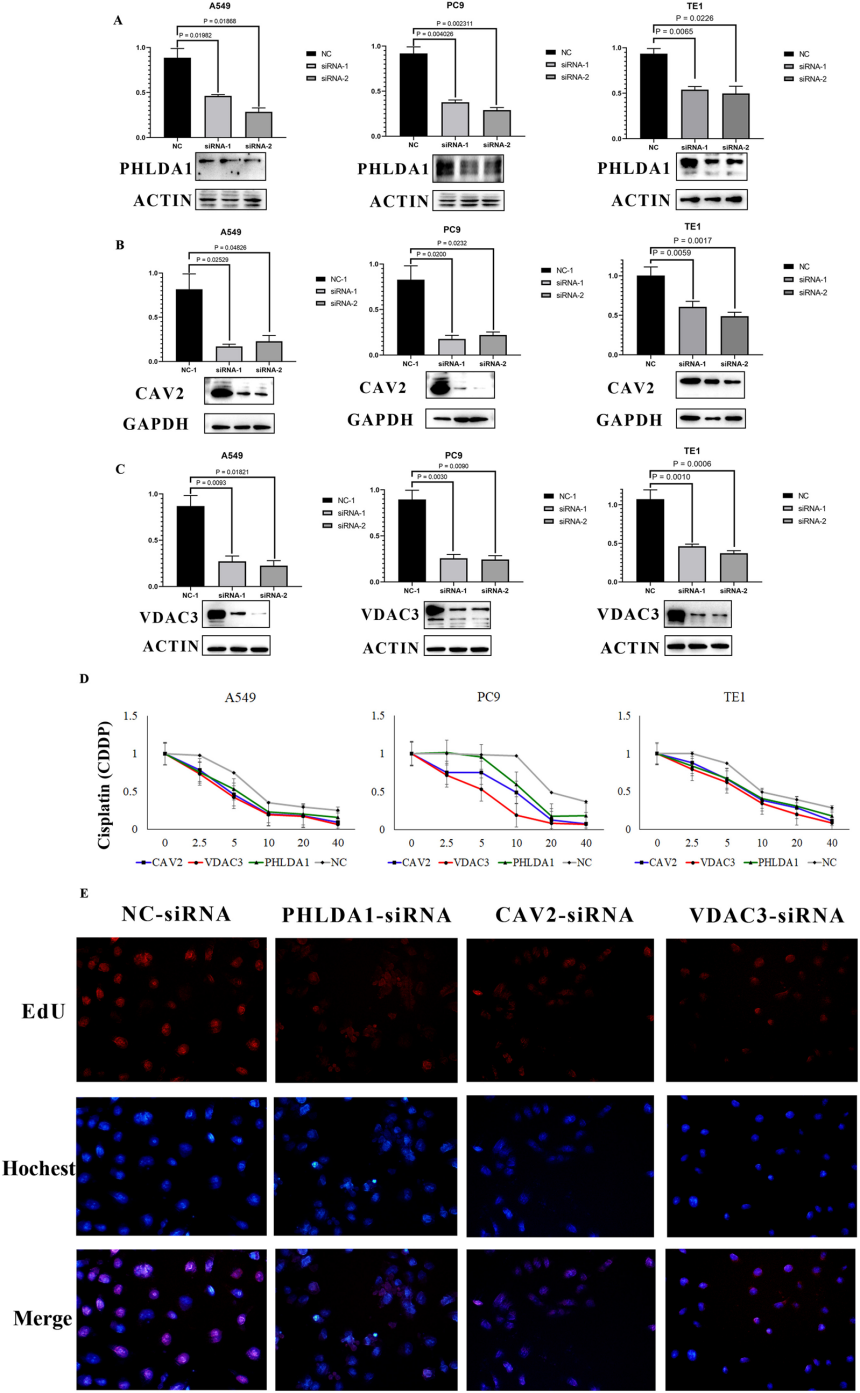
Silencing CAV2, PHLDA1, and VDAC3 separately significantly increases sensitivity to cisplatin. **A**–**C** qRT-PCR and Western blot showing CAV2, PHLDA1, and VDAC3 knockdown 48 and 72 h after transfection with CAV2-targeting, PHLDA1-targeting and VDAC3-targeting siRNAs. **D**: cytotoxicity curves of the LUAD cell lines A549, PC9 and ESCC cell line TE1 transfected with nontargeting (ctrl) or CAV2-targeting, PHLDA1-targeting and VDAC3-targeting siRNAs and treated for 48 h with different concentrations of cisplatin (0, 2.5, 5, 10, 20, 40 μM). **E** EdU for A549, PC9 and TE1 transfected with nontargeting (ctrl) or CAV2-targeting, PHLDA1-targeting and VDAC3-targeting siRNAs and treated for 48 h with cisplatin (10 μM)

Next, we validated whether the three genes most relevant to prognosis were related to cisplatin resistance at the level of in vitro experiments. We selected two LUAD cell lines, A549 and PC9, and one ESCC cell line TE1 for the experiments. First, PHLDA1, CAV2, VDAC3 and control (NC) siRNAs were transfected in the 3 cell lines to knock down the expression of these three genes (Fig. 7A–C), respectively. We then performed CCK8 and EdU assays on knockdown PHLDA1, CAV2 and VDAC3 (selected siRNA sequences with the highest knockdown efficiency and compared with knockdown NC cells) to detect changes in their sensitivity to cisplatin, respectively. As shown in Fig. 7D, E, the sensitivity of A549, PC9 and TE1 cells to cisplatin increased after the knockdown of these three genes.

## Discussion

This study evaluated the role of transcriptome gene expression in characterizing tumor subtypes, predicting chemotherapy response and long-term survival outcomes. Generally, we established a tumor sensitivity score, NCS, for platinum-containing neoadjuvant chemotherapy by analyzing single-cell data before and after neoadjuvant in LUAD and ESCC, combined with cisplatin sensitivity-related genes, as well as established a logistic prediction model based on the results of the different analysis of this score between high and low NCS groups of tumor cells in another set of single-cell samples of LUAD and ESCC, to predict the response and prognosis of platinum-containing neoadjuvant therapy.

Nearly 50% of tumor patients are treated with cisplatin [22]. Resistance to cisplatin depends on several factors, such as reduced drug accumulation and tumor cell and microenvironment heterogeneity [23]. Neoadjuvant chemotherapy (NACT) is defined as cytoreductive therapy prior to local treatment. In other words, the advancement of systemic therapy precedes local treatment. The potential benefits remain: (1) Early killing of systemic micrometastases. (2) Reducing local tumor load, decreasing tumor stage, increasing the likelihood of surgical resection and improving the rate of complete surgical resection. (3) To evaluate the effectiveness of chemotherapy in vivo to guide the correct postoperative chemotherapy; (4) To increase patient compliance and tolerability [24–27]. Even more, some patients achieve pathological complete remission (pCR) before surgery [28]; some borderline resectable advanced cancers should show a good response to treatment to achieve R0 resection. In addition, organ preservation approaches may be used for patients who have had a significant response to neoadjuvant therapy [29, 30].

However, not all patients with early or locally advanced tumors can benefit from NACT. Some patients are unable to undergo surgery due to disease progression during treatment [31]. For example, clinical research revealed that Neoadjuvant chemotherapy might exacerbate postoperative complications, which led to a negative prognostic impact [32–36]. It is therefore essential and necessary to predict the response to NAT in order to optimize treatment planning.

In our search, we started with single-cell sequenced tumor cells combined with in vitro cultured tumor cells to find the commonalities in differences in gene expression between tumor tissue before and after CDDP-NACT as well as cisplatin-sensitive and non-sensitive cell lines. Differential and enrichment analyses revealed significantly higher expression of genes related to cell adhesion, ribosome, cadherin, enzyme inhibitor, ubiquitin, etc., in the tumor cells remaining in patients after neoadjuvant therapy and cisplatin-insensitive cell lines. Actually, in previous studies, these functions were closely related to the resistance of cisplatin. For example, cell adhesion induced epithelial-mesenchymal transition, triggering a switch to a cancer-stem-cells-like phenotype and multidrug resistance [37, 38]. Cadherin may up-regulate Nanog and Sox2 to promote stemness in cancer cells, leading to chemoresistance [39, 40]. Ubiquitin has a tight relationship with the proliferation of cancer cells. It was proved that targeting USP1 (Ubiquitin-specific protease 1) ultimately increased the sensitivity of tumor cells to cisplatin and enhanced the anti-cancer efficacy [41, 42].

We obtained NCS scores as well as logistic regression models to investigate tumor heterogeneity as a critical point in cancer research, as inter- and intra-tumor variation has so far limited the development of treatment for patients. Heterogeneity in tumors already exists at the cellular level and is highly influenced by that cell's genetic background and origin and the environment in which it is established. The variety of genetic, cellular and molecular mutations is complex, which can occur during tumor development or as a response to treatment, hindering adequate clinical diagnosis and causing tumor drug resistance [43, 44]. Whereas previous studies have mainly focused on tumor tissue as a whole, thus potentially weakening the characteristics of the tumor cells themselves to some extent, the NCS score established in this study focuses on the tumor cells and can more effectively distinguish tumor subtypes that are sensitive to cisplatin-containing regimens. The presence of multiple clones within tumor cells may lead to the development of new therapies or new selections of current treatment methods toward more personalized therapy.

Furthermore, we found several vital genes, including PHLDA1, CAV2, VDAC3. Knocking down of Pleckstrin Homology-Like Domain, family A, member 1 (PHLDA1) promoting the sensitivity of cancer cells to CDDP. However, in some studies it might negatively regulate Akt activation re-sensitizing drug-resistant cancer cells to receptor tyrosine kinase (RTK)-targeted therapy [45, 46]. While in other studies, PHLDA1 overexpression promoted cell proliferation and tumor growth via Ras/Raf/Mek/Erk signaling pathway [47], indicating its complex function in tumor genesis and drug resistance. High caveolin-2 (CAV2) expression was also related to CDDP-resistance in our study. Similarly, upregulated CAV2 and the higher expression correlated with worse prognosis in PDAC [48]. And it might upregulate the proteins levels of S100s, which promotes the invasion and migration [49]. Voltage-dependent anion channels 3 (VDAC3) is upregulated in human malignant tumors, and overexpression of VDAC3 can increase sensitivity to erastin [50–52]. On the contrast, in our study, suppressing expression of VDAC3 increased cancer cell’s sensitivity to CDDP.

The present study also has some limitations. Firstly, as patients who suffered tumor progress after NACT are rarely operated on, there is a lack of post-treatment samples from these patients in this study. Future prospective matched studies could further elucidate the genomic profile of CDDP-NACT-insensitive tumor cells. Secondly, the genes used to establish NCS were used to assess the prognostic impact of platinum-containing chemotherapy in a public database of lung cancer patients. However, the lack of neoadjuvant treatment records prevented further screening. Furthermore, more studies are needed to collect more samples and combine them with samples from other malignancies treated with CDDP-NACT, such as gastric and colorectal cancers, to further refine our gene score and model, which can make it more applicable and more accurate. Finally, and most importantly, the scores and models developed in this study need to be validated in extensive prospective studies before they can be genuinely applied in clinical practice to screen patients for maximum benefit from CDDP-NACT.

## Conclusion

Through the analysis of single-cell data from LUAD and ESCC with or without NACT, cisplatin resistance-related genes obtained from the CCLE database, combined with data in various oncology public databases, we have developed NCS scores for platinum-containing neoadjuvant therapy and related predictive models, which have been validated by bioinformatic analysis as well as cell biological experiments, to assist in the clinical selection of patients who might benefit from it maximumly.

## Supplementary Information

**Additional file 1: Table S1.** Log fold change values of 12 genes in residual tumor cells after neoadjuvant chemotherapy for LUAD, ESCC, and cisplatin-resistant cell lines. **Table S2.** The sequences and melting temperatureof the primers used in our research, whether they span exon junctions. **Table S3.**

**Additional file 2: Figure S1.** UMAP showing tumor cells of LUADand ESCCwere divided into two groups of high and low expression by the mean expression of the 12 genes comprising NCS: CAV2, PHLDA1, DUSP23, VDAC3, DSG2, SPINT2, SPATS2L, IGFBP3, CD9, ALCAM, PRSS23, PERP, respectively

**Additional file 3: Figure S2.** K-M Survival analysis of 12 genes containing NCS score in LUAD patients with chemotherapy according to TCGA and GEO databases

**Additional file 4: Figure S3.** LASSO for gastric adenocarcinomaand head and neck squamous carcinomabased on TCGA. The Riskscore, survival time and survival status of selected dataset, Kaplan-Meier survival analysis of the risk model from gastric adenocarcinomaand head and neck squamous carcinoma

## Data Availability

The datasets used and/or analysed during the current study are available from the corresponding author on reasonable request.

## Abbreviations

NACT: Neoadjuvant chemotherapy
LUAD: Lung adenocarcinoma
ESCC: Esophageal squamous carcinoma
CDDP: Cisplatin
NCS: Neoadjuvant chemotherapy score
TCGA: The cancer genome atlas
CCLE: Cancer cell line encyclopedia
GEO: Gene expression omnibus
GO: Gene ontology
KEGG: Kyoto encylopaedia of genes and genomes
GSVA: Gene set variation analysis
LASSO: The least absolute shrinkage and selection operator
CCK-8: Cell counting kit-8
OD value: Optical density value
HR: Hazard ratio
CI: Confidence interval
IC50: Half maximal inhibitory concentration
