# Correction to: The genomic signature of resistance to platinum-containing neoadjuvant therapy based on single-cell data

**DOI:** 10.1186/s13578-023-01084-6

**Published:** 2023-07-21

**Authors:** Qihai Sui, Zhengyang Hu, Xing Jin, Yunyi Bian, Jiaqi Liang, Huan Zhang, Huiqiang Yang, Zongwu Lin, Qun Wang, Cheng Zhan, Zhencong Chen

**Affiliations:** Department of Thoracic Surgery, Zhongshan Hospital, Fudan University, 180 Fenglin Road, Xuhui District, Shanghai, 200032 China

**Correction: Cell & Bioscience (2023) 13:103**

10.1186/s13578-023-01061-z

In this article, the email addresses of the authors Qun Wang & Zhencong Chen was swapped.

Also, the authors Qihai Sui, Zhengyang Hu and Xing Jin should have been denoted as co-first authors.

