# Supplementary material for: The genomic signature of resistance to platinum-containing neoadjuvant therapy based on single-cell data
Source: Cell Biosci. 2023 Jun 8;13:103. doi: 10.1186/s13578-023-01061-z (PMC10249226; doi:10.1186/s13578-023-01061-z)
Supplement: Supplementary file 1 — Additional file 1: Table S1. Log fold change values of 12 genes in residual tumor cells after neoadjuvant chemotherapy for LUAD, ESCC, and cisplatin-resistant cell lines. Table S2. The sequences and melting temperatureof the primers used in our research, whether they span exon junctions. Table S3. [file 13578_2023_1061_MOESM1_ESM.docx]

**Table S1 Log fold change values of 12 genes in residual tumor cells after neoadjuvant chemotherapy for LUAD, ESCC, and cisplatin-resistant cell lines**

|  | | logFC | |
| --- | --- | --- | --- |
| Gene | LUAD | ESCC | Cisplatin (CCLE) |
| CAV2 | 0.214 | 0.370579 | 0.15313 |
| PHLDA1 | 0.26 | 0.34621 | 0.185154 |
| DUSP23 | 0.263 | 0.267914 | 0.317897 |
| VDAC3 | 0.266 | 0.438157 | 0.154416 |
| DSG2 | 0.273 | 0.451377 | 0.32043 |
| SPINT2 | 0.382427 | 0.356127 | 0.438431 |
| SPATS2L | 0.397816 | 0.402664 | 0.139543 |
| IGFBP3 | 0.439 | 1.342253 | 0.213809 |
| CD9 | 0.496336 | 1.340107 | 0.201076 |
| ALCAM | 0.531605 | 0.638841 | 0.13618 |
| PRSS23 | 0.585916 | 0.255971 | 0.192972 |
| PERP | 0.602774 | 0.455284 | 0.338851 |

**Table S2 The sequences and melting temperature (Tm) of the primers used in our research, whether they span exon junctions**

| Gene | Primers Sequence 5’-3’ (forward, reverse) | Tm (℃) | Exon junction span |
| --- | --- | --- | --- |
| CAV2 | ACGACTCCTACAGCCACCACAG | 60.0 | Yes |
|  | CCAGGAACACCGTCAGGAACTTG | 59.8 |  |
| PHLDA1 | GCATCCACATCCACATCCACACTC | 60.3 | Yes |
|  | AGTTGGAGGTGCTGCGGAGAA | 59.8 |  |
| VDAC3 | TCAGATGAGTTTTGACACAGCC | 60.5 | Yes |
|  | GAAGTCCGCAGCCTTGTAAC | 61.0 |  |

**Table S3 The sequences of the siRNAs used in our research.**

| Gene | siRNA Sequence |
| --- | --- |
| CAV2 | CAGTGCAGACAATATGGAA |
|  | GACAGATGTTATCATTGCT |
| PHLDA1 | GGAAGATGGCCCATTCAAA |
|  | TGCAGATGGTGCAGTACAA |
| VDAC3 | GAAATTGAAGGCCTCCTATAA |
|  | GGCCTCCTATAAACGGGATTG |
