# Supplementary figures and images for: The genomic signature of resistance to platinum-containing neoadjuvant therapy based on single-cell data
Source: Cell Biosci. 2023 Jun 8;13:103. doi: 10.1186/s13578-023-01061-z (PMC10249226; doi:10.1186/s13578-023-01061-z)

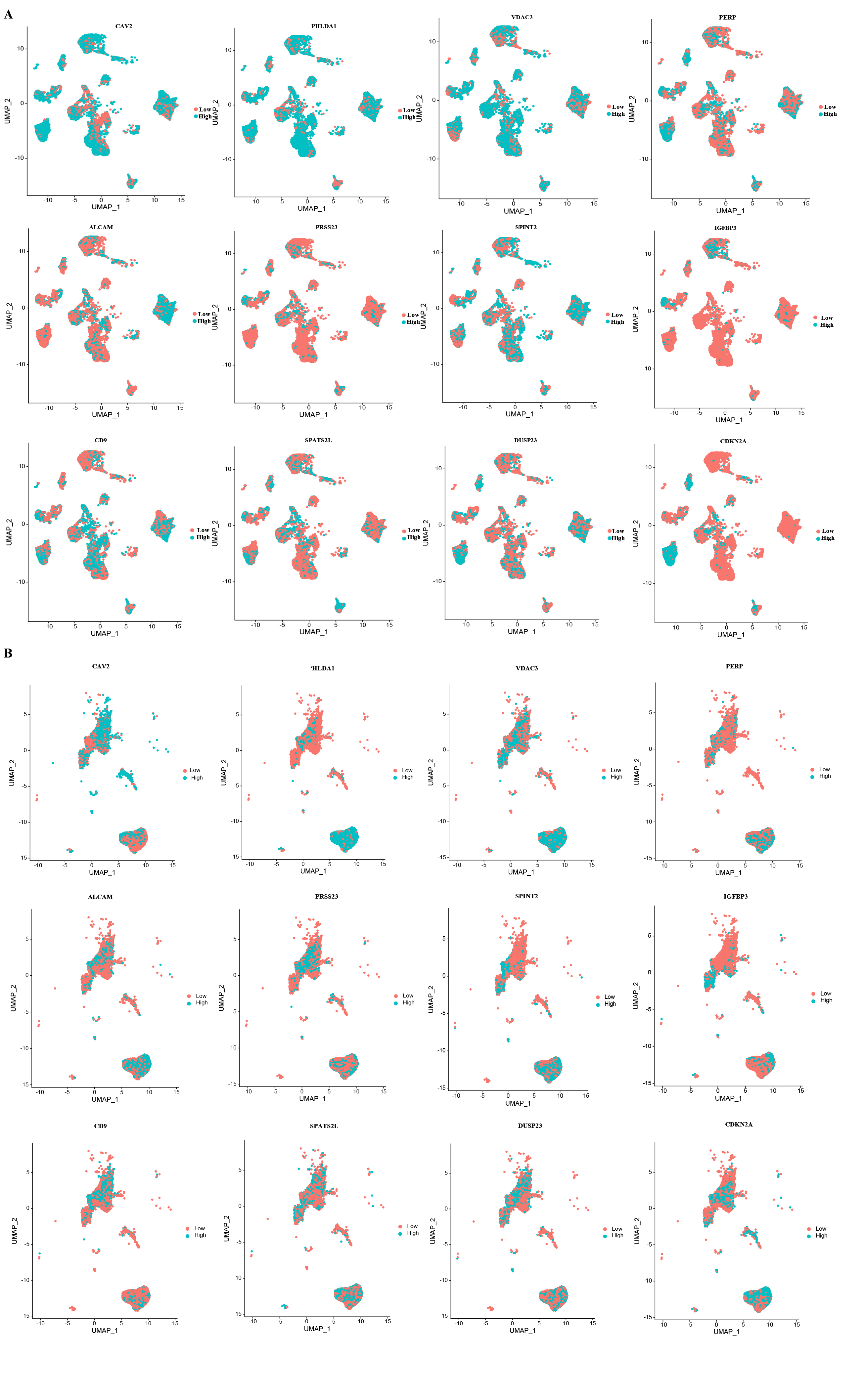

Supplement: Supplementary file 2 — Additional file 2: Figure S1. UMAP showing tumor cells of LUADand ESCCwere divided into two groups of high and low expression by the mean expression of the 12 genes comprising NCS: CAV2, PHLDA1, DUSP23, VDAC3, DSG2, SPINT2, SPATS2L, IGFBP3, CD9, ALCAM, PRSS23, PERP, respectively [file 13578_2023_1061_MOESM2_ESM.jpg]

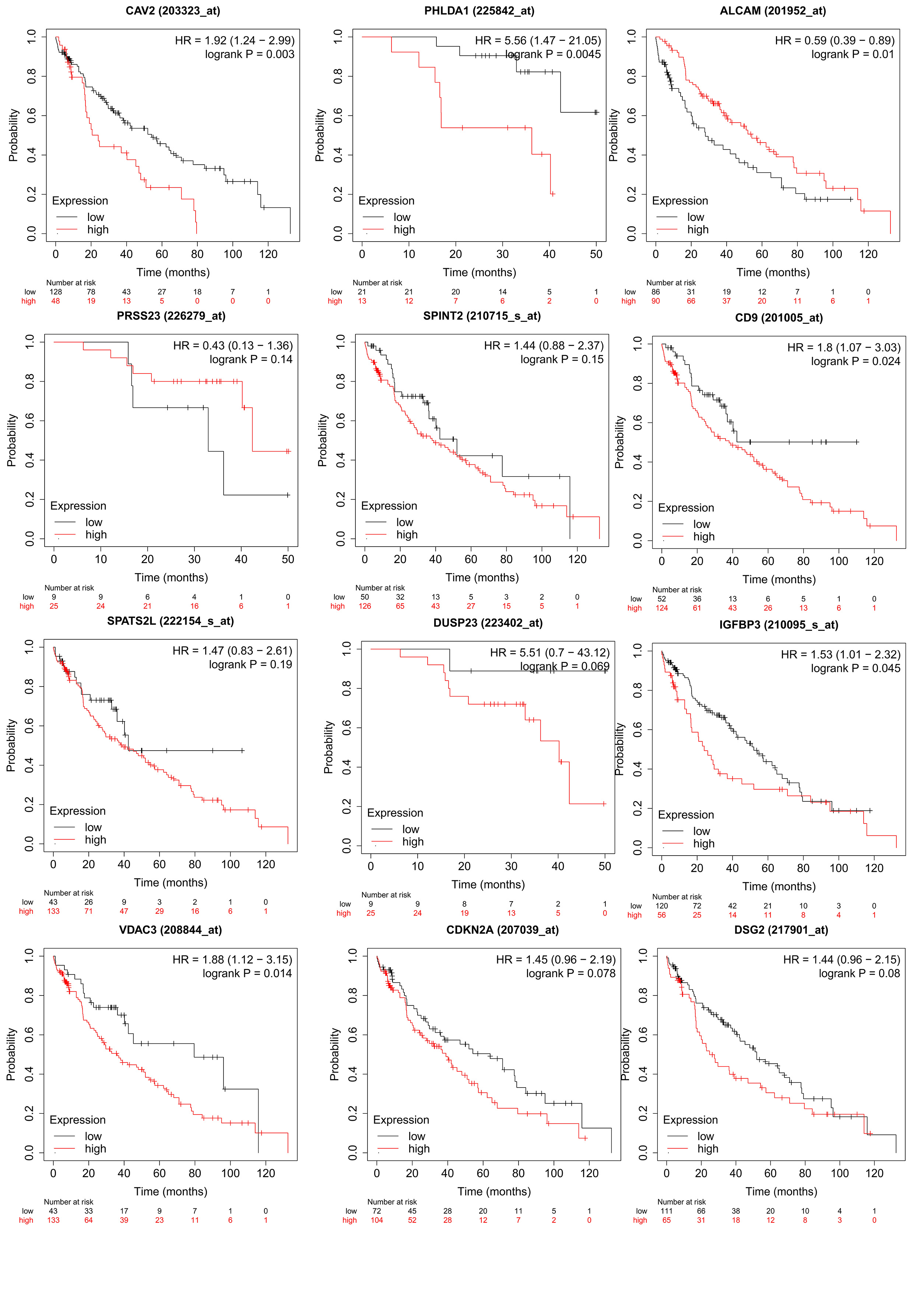

Supplement: Supplementary file 3 — Additional file 3: Figure S2. K-M Survival analysis of 12 genes containing NCS score in LUAD patients with chemotherapy according to TCGA and GEO databases [file 13578_2023_1061_MOESM3_ESM.jpg]

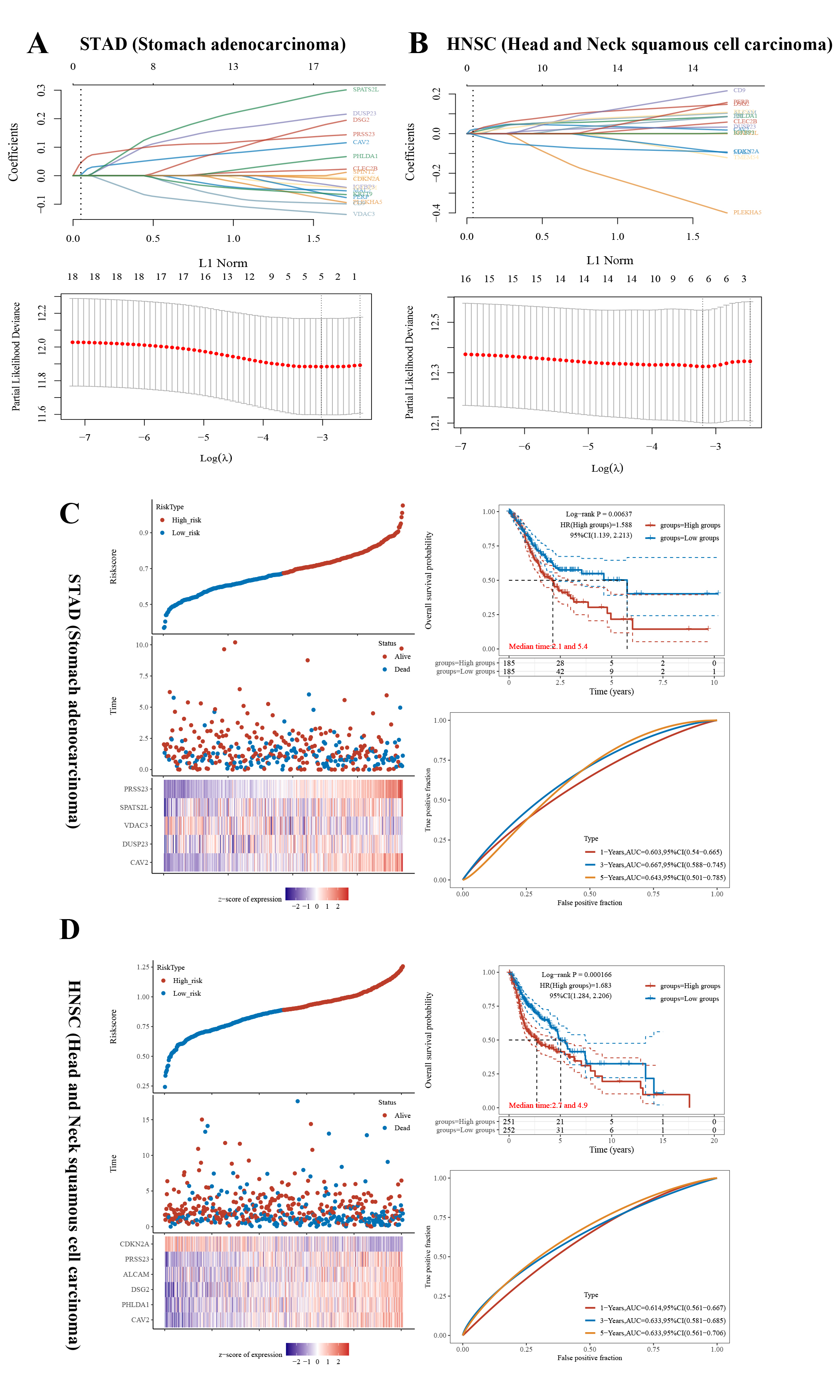

Supplement: Supplementary file 4 — Additional file 4: Figure S3. LASSO for gastric adenocarcinomaand head and neck squamous carcinomabased on TCGA. The Riskscore, survival time and survival status of selected dataset, Kaplan-Meier survival analysis of the risk model from gastric adenocarcinomaand head and neck squamous carcinoma [file 13578_2023_1061_MOESM4_ESM.jpg]
